# Chemical and nutritional characteristics, and microbial degradation of rapeseed meal recalcitrant carbohydrates: A review

**DOI:** 10.3389/fnut.2022.948302

**Published:** 2022-09-28

**Authors:** Cheng Long, Xiao-Long Qi, Koen Venema

**Affiliations:** ^1^Animal Science and Technology College, Beijing University of Agriculture, Beijing, China; ^2^Faculty of Science and Engineering, Centre for Healthy Eating and Food Innovation, Maastricht University - Campus Venlo, Venlo, Netherlands

**Keywords:** rapeseed meal, animal performance, cell wall polysaccharides, carbohydrates, gut microbial degradation

## Abstract

Approximately 35% of rapeseed meal (RSM) dry matter (DM) are carbohydrates, half of which are water-soluble carbohydrates. The cell wall of rapeseed meal contains arabinan, galactomannan, homogalacturonan, rhamnogalacturonan I, type II arabinogalactan, glucuronoxylan, XXGG-type and XXXG-type xyloglucan, and cellulose. Glycoside hydrolases including in the degradation of RSM carbohydrates are α-L-Arabinofuranosidases (EC 3.2.1.55), endo-α-1,5-L-arabinanases (EC 3.2.1.99), Endo-1,4-β-mannanase (EC 3.2.1.78), β-mannosidase (EC 3.2.1.25), α-galactosidase (EC 3.2.1.22), reducing-end-disaccharide-lyase (pectate disaccharide-lyase) (EC 4.2.2.9), (1 → 4)-6-O-methyl-α-D-galacturonan lyase (pectin lyase) (EC 4.2.2.10), (1 → 4)-α-D-galacturonan reducing-end-trisaccharide-lyase (pectate trisaccharide-lyase) (EC 4.2.2.22), α-1,4-D-galacturonan lyase (pectate lyase) (EC 4.2.2.2), (1 → 4)-α-D-galacturonan glycanohydrolase (endo-polygalacturonase) (EC 3.2.1.15), Rhamnogalacturonan hydrolase, Rhamnogalacturonan lyase (EC 4.2.2.23), Exo-β-1,3-galactanase (EC 3.2.1.145), endo-β-1,6-galactanase (EC 3.2.1.164), Endo-β-1,4-glucanase (EC 3.2.1.4), α-xylosidase (EC 3.2.1.177), β-glucosidase (EC 3.2.1.21) endo-β-1,4-glucanase (EC 3.2.1.4), exo-β-1,4-glucanase (EC 3.2.1.91), and β-glucosidase (EC 3.2.1.21). In conclusion, this review summarizes the chemical and nutritional compositions of RSM, and the microbial degradation of RSM cell wall carbohydrates which are important to allow to develop strategies to improve recalcitrant RSM carbohydrate degradation by the gut microbiota, and eventually to improve animal feed digestibility, feed efficiency, and animal performance.

## Introduction

The main ingredients of animal feeds are plant carbohydrates, which consist of more than 70% of dry matter (DM). Plant carbohydrates can be classified as non-structural carbohydrates (low molecular weight sugars, oligosaccharides, and storage polysaccharides) and structural polysaccharides (non-starch polysaccharides) (1). Non-starch polysaccharides (NSP) cannot be broken down by endogenous digestive enzymes, nor be absorbed in the upper gastrointestinal tract (GIT). However, they can be (partly) degraded by the gut microbiota in the large intestine, and absorbed by the host in the form of short-chain fatty acids (SCFA), in this manner contributing to additional energy extraction from the diet. In addition, NSP may affect the digestion of other nutrients by the means of physical hindrance or physiological changes in the gut, such as increased digesta viscosity (2). Thus, it is vital to understand the NSP composition and structure of feed ingredients to improve animal feed efficiency.

Rapeseed meal (RSM), a byproduct of rapeseed oil production, is not only a suitable protein source but also a potential energy source as animal feed. Non-structural carbohydrates make up ~3–5% of the DM. Meal production processes could affect fiber production from RSM. These include expeller pressed (rapeseed oil is physically extracted using heat), cold pressed (rapeseed oil is physically extracted without heat treatment) and solvent extraction (rapeseed oil is extracted from the meal by physical expeller extraction followed by solvent washing). Previous research showed that expeller-pressed meal had more crude fiber compared to the cold-pressed meal (3). Another research demonstrated that solvent-extraction meal had more neutral detergent fiber and acid detergent fiber compared to the cold-pressed meal (4). Non-starch polysaccharides constitute 20–40% of RSM (5, 6), which are represented by pectic polysaccharides (homogalacturonan, rhamnogalacturonan I, arabinan, galactomannan, and arabinogalactan), hemicelluloses (xyloglucan and glucuronoxylan), and cellulose (7). Reports show that the NSP can only be degraded for 3–6% in chickens (5, 8, 9), and around 58–68% in pigs. This is rather low compared to other NSP-rich feed ingredients, such as sugar beet pulp (~85% of NSP is degraded by pigs) (1). Knowledge about the relationship between the chemical structure of NSP and GIT degradation is scant.

Plant cell walls are composed of a primary and a secondary layer, which are both built from polysaccharides, lignin, and protein. Polysaccharides contribute the most to the cell wall composition. The primary cell wall of RSM contains pectin, cellulose, and xyloglucan. The cellulose microfibrils are interlinked with xyloglucan *via* hydrogen bonds forming a stiff network (10). Pectins are linked to each other and cross-linked with (Hemi)cellulose (11, 12). In the secondary cell wall of RSM, the main carbohydrates are 4-o-methyglucuronoxylan, xyloglucan, and cellulose (13). This review aims to provide an overview of the carbohydrate composition of RSM, as well as the microbial degradation of RSM. Rapeseed derives from several species belonging to the genus *Brassicaceae*. In the current review, *B. napus* is discussed.

## Nutritional characteristics of RSM carbohydrates

Carbohydrates in rapeseed meal (*B. napus*) are mainly pectins and (Hemi)celluloses and comprise 35–36% of the RSM dry matter (DM) (7, 14). The common feedstuff analysis shows commercial RSM contains 12.1% crude fiber and 34%nitrogen-freee extract (NFE) (Table 1). Carbohydrates of RSM can be categorized into non-structural carbohydrates and structural carbohydrates: a portion of the NFE is non-structural carbohydrates, whereas the crude fiber and the remainder of the NFE are structural polysaccharides. The composition of the different categories of RSM carbohydrates is displayed in Table 1.

**Table 1:** Carbohydrates composition of rapeseed meal (oil-free dry matter)^a^.

**Component, %**	**RSM**
Crude fiber	12.1
Ether extract	3.8–4.1
Acid detergent fiber	17.3
Neutral detergent fiber	22.7
Lignin	2.6
Non-structural carbohydrates^b^	3.2
Structural carbohydrates^c^	29
Nitrogen free extract	34
Monosaccharides composition, mol %	
Glucose	40
Arabinose	17
Galactose	10
Xylose	9
Fructose	1
Mannose	2
Rhamnose	2
Uronic acid	20
Water-soluble carbohydrates	18.7
Monosaccharides and sucrose	16.7
Polysaccharides	2.0
Insoluble carbohydrates	15.8
Carbohydrate total	34.5–36

^a^Adapted from references (7, 14–16).

^b^Fructose, glucose, galactose, sucrose, galactinol, raffinose and stachyose.

^c^Pectins, cellulose residue, amylose, arabinan, arabinogalactan, xyloglucan.

### Non-structural carbohydrates

**Figure 1:**
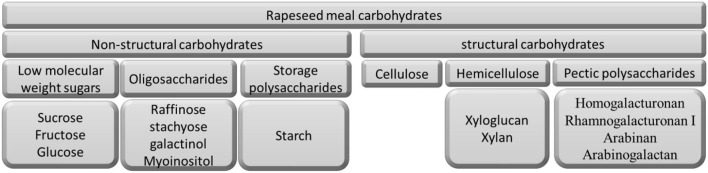
Schematic representation of carbohydrates presented in rapeseed meal.

The non-structural carbohydrates in RSM are comprised of low molecular weight sugars, oligosaccharides, and storage polysaccharides (Figure 1).

#### Low molecular weight sugars

Low molecular weight sugars mainly are sucrose (2.3–2.9%), fructose (0.05–0.16%), and glucose (0.05–0.16%) (15).

#### Oligosaccharides

The primary oligosaccharides found in RSM are raffinose, stachyose, galactinol, and Myo-inositol. Of those, stachyose has the highest concentration (0.4–0.5%), followed by raffinose (0.05–0.16%), galactinol (0.1%), and Myo-inositol (0.1%) (6).

#### Storage polysaccharides

Only low concentrations of storage polysaccharides are present in RSM. The primary storage polysaccharide is starch. The starch content of the seed approaches 50% during early development, but starch almost completely disappears when energy stores are converted into oil as the seed matures. Rommi et al. (14) reported that starch concentrations found in the intact seed were the same as dehulled rapeseed press cakes (0.2% of DM), while the starch content of canola meal was up to 2.5% of DM (17). Starch granules are comprised of two main macromolecules: amylose and amylopectin. Amylose is a linear polymer consisting of α-1,4-linked D-glucose units (GU) and includes 500–600 GU in its native form, which can be subdivided into 1–20 chains. Amylopectin is a large branched macromolecule (molecular weight 10^7^-10^9^ kDa), with ~5% of its total linkages consisting of β-1,6-linked GU plus a large number of short α-1,4-linked linear GU chains (12–70 GU) (18).

### Structural polysaccharides

The largest part of the RSM carbohydrates is made up of structural polysaccharides (Figure 1). These include cellulose, hemicellulose (xyloglucan and xylan), and pectic polysaccharides (homogalacturonan, rhamnogalacturonan I, arabinan, and arabinogalactan).

#### Polysaccharides composition

RSM contains 34.5% NSP of its DM, and RSM is high in glucosyl (40 mol%), arabinosyl (19 mol%), and uronyl residues (18 mol%) (7, 19). Water-soluble and water-insoluble carbohydrates comprise 18.7 and 15.8% of the total carbohydrates content of RSM, respectively (Table 1). Water-soluble carbohydrates mainly contain glucosyl (64 mol%) and some galactosyl residues (17 mol%) (7), while water-insoluble carbohydrates are mainly glucosyl (32 mol%), arabinosyl (25 mol%), uronyl (18 mol%), and xylosyl residues (12%) (Table 2).

**Table 2:** Molar sugar composition of RSM fractions^a^.

	**Molar composition (mol%)**						
	**Rha**	**Ara**	**Xyl**	**Man**	**Gal**	**Glc**	**UA**
RSM	2	19	8	6	10	40	15
WSS	Tr^b^	7	1	7	17	64	5
WUS	1	25	12	4	8	32	18
CHSS	2	15	4	2	4	3	71
DASS	Tr	44	6	3	10	9	29
4MASS	Tr	17	20	15	13	30	5
6MASS	1	29	22	2	13	23	11
RES	4	10	6	4	6	40	20

^a^Adapted from reference (7).

^b^Trace amounts.

**Figure 2:**
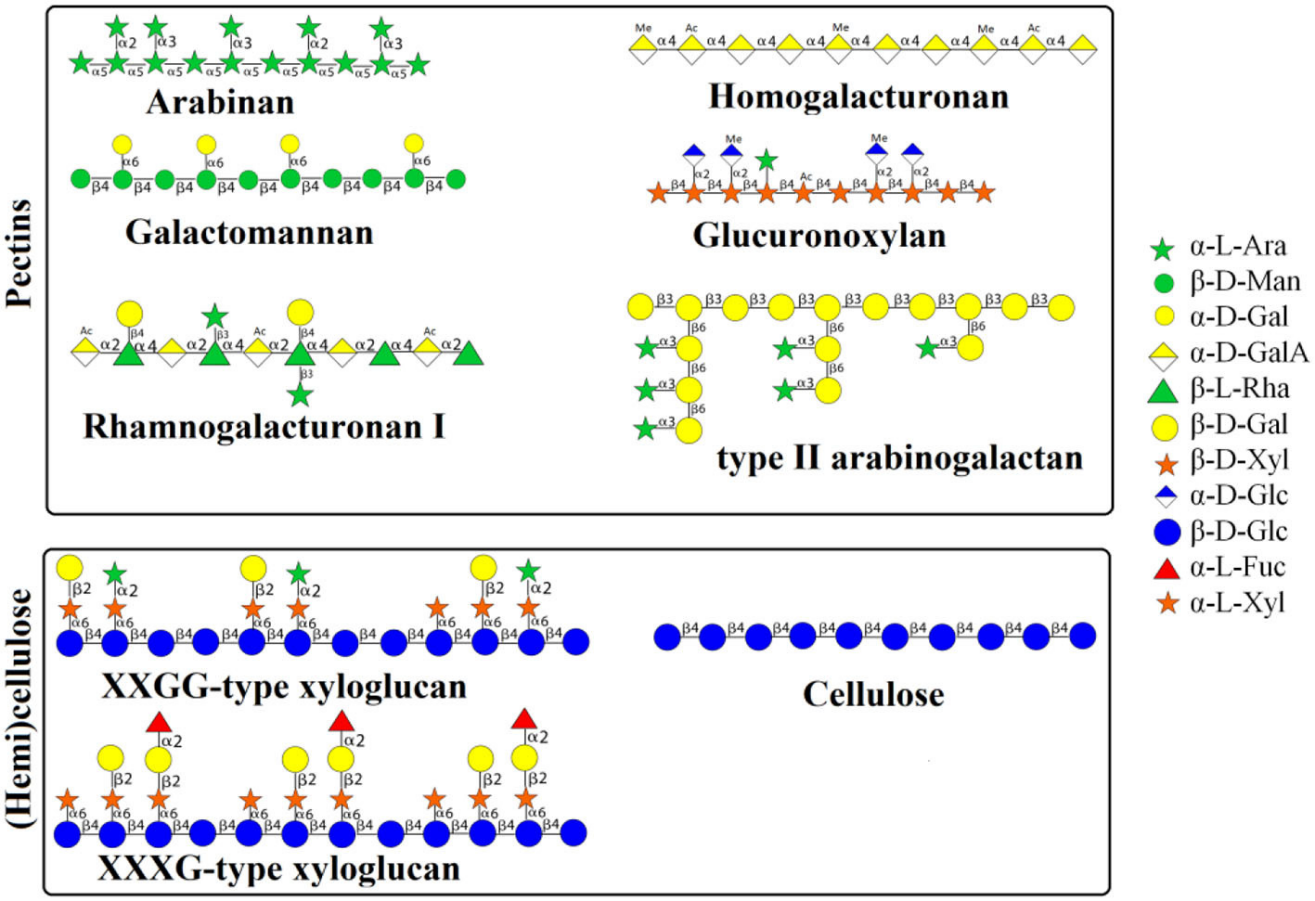
Schematic representation of structures of polysaccharides in RSM. RSM, rapeseed meal; WSS, water soluble solids; WUS, water unextractable solids; ChSS, Chelating Agent Soluble Solids; DASS, Dilute Alkali Soluble Solids; 4 MASS, 4 Molar Alkali Soluble Solids; 6 MASS, 6 Molar Alkali Soluble Solids; RES, residue; Ara, arabinose; Xyl, xylose; Man, mannose; Fuc, fucosyl; Gal, galactose; Glc, glucose; UA, uronic acids.

The detailed monosaccharide constituent compositions of the Water unextractable solids (WUS), which were sequentially extracted with (i) chelating agent (ChSS) to release calcium-bound pectins, (ii) dilute alkali (DASS) to release pectins tightly bound to hemicellulose, (iii) 4, and (iv) 6 molar alkali (4 MASS and 6 MASS) to release hemicelluloses, leaving cellulose that remains in the residue (RES), are presented in Table 2. The composition of the glycosidic residues indicates the presence of α-1,5-linked arabinan branched at O-2, galactomannan, homogalacturonan, rhamnogalacturonan I, type II arabinogalactan, glucuronoxylan, XXGG-type and XXXG-type xylo(X)glucan(G), and cellulose in RSM (7, 14, 20). Arabinan consists of a linear backbone of α-L-1,5-linked arabinose (Ara) units with α-1,2-linked or α-1,3-linked Ara units (Figure 2). Galactomannan has a backbone of β-1,4-linked mannose units, substituted with α-1,6-linked galactose units (Figure 1). Homogalacturonan (HG) is the simplest form of pectin, consisting of a linear chain of α-1,4-linked galacturonic acid, and part of its carboxyl groups esterified with acetyl or methyl groups (Figure 2). Rhamnogalacturonan I (RG-I) consists of alternating rhamnose (Rha) and galacturonic acid (GalA) residues [-,2)-α-Rha-(1,4)-α-D-GalA-(1,-], which is highly ramified with single terminal β-D-Gal and/or α-D-Ara at position O-4 or O-3 of the rhamnosyl residues where the α-D-GalA residues are often O-acetyl esterified at O-2 and/or O-3 (Figure 2) (21). Type II arabinogalactan (AG) is composed of a β-1,3-galactan backbone and β-1,6-galactan side chains (Figure 2). The side chains are variably decorated with L-arabinose. Glucuronoxylan (GX) has a linear backbone of β-(1-4)-linked D-xylosyl (Xyl) residues, which can be ramified with acetyl and arabinosyl residues, and some of the Xyl residues are decorated with a single α-D-glucuronic acid (GlcA) or 4-O-methyl-D-glucuronic acid (MeGlcA) residue at O2 (Figure 2) (22, 23). Both XXGG- and XXXG-type xyloglucan (XG) exist in RSM. XXGG (Figure 2) consists of a β-1,4-linked D-glucosyl (Glc) backbone carrying various side chains of D-β-1,2-Gal-D-α-1,6-Xyl and L-α-1,2-Ara-D-β- D-α-1,6-Xyl (24), whereas, XXXG (Figure 2) comprises a β-1,4-linked D-glucosyl backbone carrying continuous side chains of D-α-1,6-Xyl, D-β-1,2-Gal-D-α-1,6-Xyl, and L-α-1,2-Ara-D-β-1,2-Gal-D-α-1,6-Xyl in every 4 residues of D-β-1,4-Glc (24). Cellulose consists of long linear chains of β-1,4-linked D-Glc residues, with a degree of polymerization between 2,000 and 14,000 residues (Figure 2). The backbone of these polysaccharides can also be esterified with methyl-esters, ethyl-esters, and glycosyls (arabinosyl, galactosyl, mannosyl, gucosyl, xylosyl, or frucosyl).

Except for containing glucosyl residues, RES (Table 2) also has quite some arabinosyl and uronyl residues (10 and 20%, respectively), which indicates that some pectic polysaccharides are tightly associated with cellulose microfibrils. This suggests that the cell wall polysaccharide matrix of rapeseed meal is strongly interlinked. The specific structures of dietary fibers of RSM are still not entirely understood. The main restriction may be the analysis method, but nowadays, the comprehensive microarray polymer profiling (CoMPP) technique is a powerful tool for probing cell wall structure studies (25). The profiles generated by CoMPP provide a global snapshot of cell-wall composition. It cannot only detect the amount of the particular polysaccharides but also their linkage type as discussed in the next section.

#### Glycosidic linkage type

A covalent link between one carbohydrate molecule and a second carbohydrate molecule is called a glycosidic bond. Glycosidic linkage is the type of bond between two adjacent glycosides in the chain of polysaccharides (26).

The proposed sugar linkage compositions of RSM fractions are shown in Table 3. The data can only be used in a qualitative manner instead of quantitative, due to the poor DMSO solubility of the fractions and the high amount of uronic acids (which are not detected with this method) present in some samples (27).

**Table 3:** Sugar linkage composition of RSM fractions (mol%)^a^.

	**WUS**	**DASS**	**4 MASS**	**6 MASS**
t-Ara^b^	18	41	16	15
1,2-Ara	–	1	1	–
1,5-Ara	10	9	2	11
1,2,5-Ara	6	29	10	4
Total Ara	34	80	29	30
t-Xyl	9	3	7	12
1,2-Xyl	2	1	10	3
1,4-Xyl	3	1	3	7
Total Xyl	14	5	20	22
1,4,6-Man	–	–	3	1
Total Man	–	–	3	1
t-Fuc	2	1	2	5
1,2,4-Fuc	7	3	–	8
Total Fuc	9	4	2	13
t-Gal	3	–	4	6
1,2-Gal	–	–	5	7
1,3-Gal	–	–	7	–
1,3,6-Gal	–	7	4	–
Total Gal	3	7	20	13
1,4-Glc	25	5	17	6
1,4,6-Glc	16	–	11	15
Total Glc	41	5	28	21
T/B^c^	1.10	1.15	1.04	1.36

^a^Adapted from reference (14).

^b^t, terminal.

^c^T/B, ratio terminally linked residues: branching points.

Ara, arabinose; Xyl, xylose; Man, mannose; Fuc, fucose; Gal, galactose; Glc, glucose; UA, uronic acid.

## Microbial degradation of RSM cell wall carbohydrates

Low molecular weight sugars and starch can be 100% digested and/or absorbed, while (oligo- and poly-)saccharides are considered indigestible in the small intestine due to the lack of the necessary enzymes in monogastric animals (8). Indeed, mammalian genomes do not encode most of the enzymes needed to degrade the structural polysaccharides present in plant material. Instead, a complex mutual dependence has developed between the mammalian host and symbiotic gut microorganisms that do possess the ability to access the abundant source of energy in carbohydrates that are indigestible by the host. The gut microbiota has glycoside hydrolases (GH) that can degrade the oligo- and polysaccharides into small oligomers and monosaccharides which are subsequently taken up and fermented.

### Glycoside hydrolases

Glycoside hydrolases (GHs) are a vast repertoire of cell wall-degrading enzymes that hydrolyze glycosidic bonds between two or more carbohydrate modules or sugar and a non-sugar moiety within carbohydrates or oligosaccharides (28, 29). GH families widely exist in prokaryotic, eukaryotic, and archaea species. A total of 173 GH families have been identified until now (accessed on May-2022, http://www.cazy.org/Glycoside-Hydrolases.html).

Carbohydrate binding modules (CBMs) are the non-catalytic part of cell-wall-degrading enzymes, and they are attached to the GH catalytic modules. Usually, CBMs have to recognize and bind to the specific polysaccharides first, before the GHs cleave the polysaccharides (30).

### Degradation of arabinan

Arabinan can be hydrolyzed by α-L-Arabinofuranosidases (EC 3.2.1.55) and endo-α-1,5-L-arabinanases (EC 3.2.1.99), which are found in GH families 3, 43, 51, 54, and 62, and which release arabinosyl oligomers and L-arabinose (31). A previous study reported that *Bacillus subtilis* contained a series of arabinan-degrading genes, *abnA, abn2, abfA*, and *abf2*, which were induced by arabinose and arabinan, repressed by glucose, and subjected to temporal regulation (32). *AbnA* and *abn2* encode extracellular endo-α-1,5-L-arabinanases belonging to GH43, which hydrolyzes arabinan (branched) and linear α-1,5-L-arabinan and produces arabinose and arabino-oligosaccharides (33). These resulting products, are subsequently transported into the cell by different transport systems. Arabinose enters the cell mainly through the *araE* permease (34), and the uptake of arabinose oligomers occurs most likely *via araNPQ*, an ABC-type transporter (35). These products are further digested into the monosaccharide arabinose by two intracellular arabinofuranosidases, *abfA* and *abf2*, which are α-L-arabinofuranosidases (EC 3.2.1.55) belonging to GH51. *AbfA* acts preferentially on (1 → 5) arabinofuranosyl linkages, and in contrast, *abf2* is most active on (1 → 2) and (1 → 3) linkages (36). After this, L-arabinose is converted into D-xylulose-5-phosphate, which is further catabolized through the pentose phosphate pathway. The induction mechanism of these genes is mediated through negative control by the key regulator of arabinose metabolism, *araR*. The transcriptional repression of the *abfA* and *abf2* genes is achieved by a tightly controlled mechanism but the regulation of *abnA* is more flexible.

The presence of α-L-arabinofuranosidases (Table 4) has also been determined in *Bifidobacterium longum* subsp. *longum* (37), *Bacteroides thetaiotaomicron* VPI-5482 (38), *Sulfolobus solfataricus* P2 (39), *Anoxybacillus kestanbolensis* AC26Sari (40), *Monoglobus pectinilyticus* (41), and *Roseburia faecis* M72/1 (42). Endo-α-1,5-L-arabinanases (Table 4) are present in *Paenibacillus polymyxa* (43), *Bacillus licheniformis* (44), *Caldicellulorsiruptor saccharolyticus* (45), *Bacillus subtilis* (36), *Bacillus thermodenitrificans* (46), *Pseudomonas fluorecens* subsp. *cellulosa* (47), *Monoglobus pectinilyticus* (41), and *Roseburia faecis* M72/1 (42).

**Table 4:** Gut microbes containing the indicated plant cell wall degrading enzymes.

**Microbial enzymes**	**Microorganism**
α-L-arabinofuranosidases	*Bifidobacterium longum* subsp. *longum* (37), *Bacteroides thetaiotaomicron* VPI-5482 (38), *Sulfolobus solfataricus* P2 (39), *Anoxybacillus kestanbolensis* AC26Sari (40), *Monoglobus pectinilyticus* (41), and *Roseburia faecis* M72/1 (42)
endo-α-1,5-L-arabinanases	*Paenibacillus polymyxa* (43), *Bacillus licheniformis* (44), *Caldicellulorsiruptor saccharolyticus* (45), *Bacillus*. *Subtilis* (36), *Bacillus*. *Thermodenitrificans* (46), *Pseudomonas fluorecens* subsp. *cellulosa* (47), *Monoglobus pectinilyticus* (41), and *Roseburia faecis* M72/1(42)
endo-1,4-β-mannanase	*Bacillus subtilis* YH12 (49), *Bacillus subtilis* TD7 (50), *Bacillus subtilis* Bs5 (51), *Bacillus licheniformis* DSM13 (52), *Sphingomonas* sp. JB13 (53), *Sphingobacterium* sp. GN25 (54), *Klebsiella oxytoca* KUB-CW2-3 (55), *Enterobacter* sp. *strain* N18 (56), *Flavobacterium* sp. (57), *Pseudomonas cellulosa* (47), *Monoglobus pectinilyticus* (41), and *Bacteroides ovatus* (58)
β-mannosidase	*Bifidobacterium* sp. (59), *Bifidobacterium longum* subsp. *longum* NCC2705 (60), *Bacteroides ovatus* (61, 62), *Cellvibrio mixtus* (63), *Bacteroides thetaiotaomicron* (64), *Pseudomonas cellulose* (47), *Kitasatospora* sp. (65)
α-Galactosidases	*Lactobacillus acidophilus* NCFM (66), *Lactobacillus crispatus* ST1 (67), *Lactobacillus brevis* (68), *Bifidobacterium animalis* subsp. *lactis* Bl-04 (66), *Arthrobacter* sp. C2-2 (69), *Bacillus megaterium* (70), *Dictyoglomus thermophilum* (71), *Bacillus stearothermophilus* NCIM-5146 (72), *Bacillus stearothermophilus* NUB 3621 (73), *Bacteroides ovatus* 0038-1 (74), *?ifidobacterium bifidum* NCIMB41171 (75), *?ifidobacterium adolescentis* DSM20083 (76), *?ifidobacterium breve* 203 (77), *Clostridium stercorarium* (78), and *Monoglobus pectinilyticus* (41)
Pectate disaccharide-lyase	*Caldicellulosiruptor bescii* (81), *Bacteroides thetaiotaomicron* (82), *Bacteroides ovatus* (83), *Bacillus pumilus* BK2 (84), *Eubacterium eligens, Faecalibacterium prausnitzii* (85), and *Monoglobus pectinilyticus* (41)
pectate lyase	in *Caldicellulosiruptor bescii* (81), genus *Bacillus: Bacillus subtilis, B. licheniformis, B. cereus, B. circulans, B. pasteurii*, B. *amyloliquefaciens*, and *B. pumilus* (86, 87), *Paenibacillus* sp. (88), *Clostridium cellulovorans* (89), *Streptomyces thermocarboxydus* (90), *Bacteroides thetaiotaomicron* (82), *Bacteroides ovatus* (83), *Eubacterium eligens, Faecalibacterium prausnitzii* (85), and *Monoglobus pectinilyticus* (41)
endo-polygalacturonase	*Caldicellulosiruptor bescii* (81), *Bacteroides thetaiotaomicron* (82), *Bacteroides ovatus* (83), *Eubacterium eligens, Faecalibacterium prausnitzii* (85), *Monoglobus pectinilyticus* (41), and *Bifidobacterium longum* subsp. *longum* (91)
rhamnogalacturonan lyase and hydrolase	*Caldicellulosiruptor bescii* (81), *Bacteroides thetaiotaomicron* (82), *Bacteroides ovatus* (83), *Bacillus subtilis* (92), *Bacillus licheniformis* (93), *Cellvibrio japonicus* (94), *Clostridium cellulolyticum* (95), *Bacillus licheniformis* DSM13 (93), *Pseudomonas cellulose* (94), *Penicillium chrysogenum* (96), and *Monoglobus pectinilyticus* (41)
exo-β-1,3-galactanase	*Monoglobus pectinilyticus* (41), *Bifidobacterium longum* subsp. *longum* (91), *Clostridium thermocellum* (97), *Phanerochaete chrysosporium* (98), *Sphingomonas* sp. (99), *Bacteroides thetaiotaomicron* (100), *Bacteroides ovatus* (100), *Bacteroides caccae* (101), and *Bacteroides cellulosilyticus* (101)
endo-β-1,6-galactanase	*Streptomyces avermitilis* NBRC14893 (102), *Bacteroides ovatus* (100), *Bacteroides caccae* (101), *Bacteroides cellulosilyticus* (101), and *Bifidobacterium longum* subsp. *longum* (91)
exo-β-1,6-galactobiohydrolase	*Monoglobus pectinilyticus* (41), *Bacteroides thetaiotaomicron* (101), *Streptomyces avermitilis* (102), *Bacteroides ovatus* (100), *Bacteroides caccae* (101), *Bacteroides cellulosilyticus* (101), and *Bifidobacterium longum* subsp. *longum* (91)
endo-β-1,4-xylanase	*Pseudomonas boreopolis* G22 (103), *Bacteroides ovatus* (82), *Monoglobus pectinilyticus* (41), *Bacteroides thetaiotaomicron* (100), *Bacteroides caccae* (101), *Bacteroides cellulosilyticus* (101), *Clostridium thermocellum* (104), *Bacillus subtilis* (105), and *Streptomyces turgidiscabies* (106)
exo-β-1,4 xylanase	*Monoglobus pectinilyticus* (41), *Luteimicrobium* xylanilyticum (107), *Amycolatopsis mediterranei* (107), *Clostridium thermocellum* (104), *Bacillus subtilis* (105), and *Streptomyces turgidiscabies* (106)
endo and exo-β-1,4-glucanase	*Monoglobus pectinilyticus* (41), *Caldicellulosiruptor kronotskyensis* (108), *Roseburia* sp. (42), *Eubacterium rectale* group (42), *Ruminococcus champanellensis* (109), *Ruminococcus bromii* (110), *Ruminiclostridium cellulolyticum* (111), and *Phaeoacremonium minimum* (112)
α-D-xylosidase	*Sulfolobus solfataricus* P2 (39), *Talaromyces thermophilus* (113), *Cellvibrio japonicus* (114), *Bacteroides thetaiotaomicron* (82), *Bacteroides ovatus* (82), and *Monoglobus pectinilyticus* (41)
β-glucosidase	*Bifidobacterium adolescentis* (115), *Bacteroides ovatus* (116), *Listeria innocua* (117), *Streptomyces venezuelae* (118), *Pyrococcus furiosus* (119), *Cellvibrio japonicus* (114), *Caldicellulosiruptor saccharolyticus* (120), *Microbispora bispora* (121), *Thermoanaerobacter brockii* (122), *Thermobifida fusca* (103), *Pseudomonas* sp. (123), *Monoglobus pectinilyticus* (41), *Ruminococcus champanellensis* (109), and *Ruminococcus bromii* (110)

### Degradation of galactomannan

Endo-1,4-β-mannanase (EC 3.2.1.78), β-mannosidase (EC 3.2.1.25) and α-galactosidase (EC 3.2.1.22) are involved in the degradation of galactomannan into monosaccharides (48). β-mannanase degrades randomly within the main chain of galactomannans and produces shorter galactomanno-oligosaccharides that can be further hydrolyzed by β-mannosidase and α-galactosidase. β-mannosidase hydrolyses β-1,4-linked mannose residues from the non-reducing end of the galactomanno-oligosaccharides and α-galactosidase hydrolyses terminal α-1,6-linked galactose residues from galactomannans or the galactomanno-oligosaccharides. The gut microbes *Bacillus subtilis* YH12 (49), *Bacillus subtilis* TD7 (50), *Bacillus subtilis* Bs5 (51), *Bacillus licheniformis* DSM13 (52), *Sphingomonas* sp. JB13 (53), *Sphingobacterium* sp. GN25 (54), *Klebsiella oxytoca* KUB-CW2-3 (55), *Enterobacter* sp. *strain* N18 (56), *Flavobacterium* sp. (57), *Pseudomonas cellulosa* (47), *Monoglobus pectinilyticus* (41), and *Bacteroides ovatus* (58) are reported to have endo-1,4-β-mannanase (Table 4). While *Bifidobacterium* sp. (59), *Bifidobacterium longum* subsp. *longum* NCC2705 (60), *Bacteroides ovatus* (61, 62), *Cellvibrio mixtus* (63), *Bacteroides thetaiotaomicron* (64), *Pseudomonas cellulose* (47), and *Kitasatospora* sp. (65) are reported to have β-mannosidase (Table 4). Moreover, *Lactobacillus acidophilus* NCFM (66), *Lactobacillus crispatus* ST1 (67), *Lactobacillus brevis* (68), *Bifidobacterium animalis* subsp. *lactis* Bl-04 (66), *Arthrobacter* sp. C2-2 (69), *Bacillus megaterium* (70), *Dictyoglomus thermophilum* (71), *Bacillus stearothermophilus* NCIM-5146 (72), *Bacillus stearothermophilus* NUB 3621 (73), *Bacteroides ovatus* 0038-1 (74), *?ifidobacterium bifidum* NCIMB41171 (75), *?ifidobacterium adolescentis* DSM20083 (76), *?ifidobacterium breve* 203 (77), *Clostridium stercorarium* (78), and *Monoglobus pectinilyticus* (41) are reported to have α*-*galactosidases (Table 4).

*Asperigillus niger* contains galactomannan-degradation genes, *aglA, algB*, and *algC* (encoding α-galactosidases), and *mndA* (encoding a β-mannosidase). *AglA* and *aglB* have been classified into GH27, while *aglC* has been classified into GH36, and mndA belongs to GH2. The metabolism mechanism of galactomannan is that α-galactosidase (encoded by *aglC*) and β-mannosidase (encoded by *mndA*) hydrolyze the galactomannan to the oligosaccharide Gal2Man5, where *mndA* cleaves single mannose units from the non-reducing end of the substrate until it reaches a galactose side-group (79), and afterward the non-reducing galactose group is hydrolyzed by α-galactosidase (*aglA, aglB*, or *aglC*). *AglB* and *mndA* play a major role in the degradation of galactomannan in *A. niger*. The expression of *aglA* is high on galactose and galactose-containing oligosaccharides, but is fully repressed in the presence of glucose (80). Little is known about genes, and especially their regulation, in members of the gut microbiota.

### Degradation of homogalacturonan

Homogalacturonan (HG) can be cleaved by α-1,4-L-galacturonan reducing-end-disaccharide-lyase (pectate disaccharide-lyase) (EC 4.2.2.9), (1 → 4)-6-O-methyl-α-D-galacturonan lyase (pectin lyase) (EC 4.2.2.10), (1 → 4)-α-D-galacturonan reducing-end-trisaccharide-lyase (pectate trisaccharide-lyase) (EC 4.2.2.22), α-1,4-D-galacturonan lyase (pectate lyase) (EC 4.2.2.2), and (1 → 4)-α-D-galacturonan glycanohydrolase (endo-polygalacturonase) (EC 3.2.1.15) (89, 124–128). Pectin lyase (EC 4.2.2.10) provides cleavage of α-1,4-linked D-galacturonan methyl ester to give oligosaccharides with 4-deoxy-6-O-methyl-α-D-galact-4-enuronosyl groups at their non-reducing ends, while α-1,4-D-galacturonan lyase (EC 4.2.2.2) cleaves α-1.4-linked D-galacturonan to give oligosaccharides with 4-deoxy-α-D-galact-4-enuronosyl groups at their non-reducing ends. Afterwards, pectate disaccharide-lyase (EC 4.2.2.9) hydrolyzes these oligosaccharides to (1,4-α-D-galacturonosyl)_n − 2_ and 4-(4-deoxy-α-D-galact-4-enuronosyl)-D-galacturonate, and (1,4-α-D-galacturonosyl)_n − 2_ will be cleaved by pectin lyase (EC 4.2.2.10 or EC 2.2.2) again until the disaccharide results. Polygalacturonase (EC 3.2.1.15) randomly hydrolyzes (1 → 4)-α-D-galactosiduronic linkages in pectate and other galacturonans. Pectate disaccharide-lyase (Table 4) has been reported in *Caldicellulosiruptor bescii* (81), *Bacteroides thetaiotaomicron* (82), *Bacteroides ovatus* (83), *Bacillus pumilus* BK2 (84), *Eubacterium eligens, Faecalibacterium prausnitzii* (85), and *Monoglobus pectinilyticus* (41). Pectate lyase (Table 4) has been reported in *Caldicellulosiruptor bescii* (81), genus *Bacillus: Bacillus subtilis, B. licheniformis, B. cereus, B. circulans, B. pasteurii, B*. *amyloliquefaciens*, and *B. pumilus* (86, 87), *Paenibacillus* sp. (88), *Clostridium cellulovorans* (89), *Streptomyces thermocarboxydus* (90), *Bacteroides thetaiotaomicron* (82), *Bacteroides ovatus* (83), *Eubacterium eligens, Faecalibacterium prausnitzii* (85), and *Monoglobus pectinilyticus* (41). Endo-polygalacturonase (Table 4) has been reported in *Caldicellulosiruptor bescii* (81), *Bacteroides thetaiotaomicron* (82), *Bacteroides ovatus* (83), *Eubacterium eligens, Faecalibacterium prausnitzii* (85), *Monoglobus pectinilyticus* (41), and *Bifidobacterium longum* subsp. *longum* (91).

### Degradation of rhamnogalacturonan I

Utilization of rhamnogalacturonan I (RG-I) by microbes is mediated by a series of enzymes, which is well-studied in *Bacillus subtilis* (92). Two main enzymes, a hydrolase, and a lyase are involved in the degradation of the RG-I backbone, whereas a few other enzymes are responsible for the breakdown of the RG-I side chains. Rhamnogalacturonan hydrolase (rhamnogalacturonan α-L-rhamnopyranohydrolase) cleaves α-1,2 linkages between GalA and Rha (129). Rhamnogalacturonan lyase [-L-rhamnopyranosyl-(1 → 4)-alpha-D-galactopyranosyluronate endolyase (EC 4.2.2.23)] cleaves the α-1,4 linkages of RG-I resulting in a double bond in the on-reducing GalA residue (130). Rhamnogalacturonan lyase and hydrolase (Table 4) have been reported in *Caldicellulosiruptor bescii* (81), *Bacteroides thetaiotaomicron* (82), *Bacteroides ovatus* (83), *Bacillus subtilis* (92), *Bacillus licheniformis* (93), *Cellvibrio japonicus* (94), *Clostridium cellulolyticum* (95), *Bacillus licheniformis* DSM13 (93), *Pseudomonas cellulose* (94), *Penicillium chrysogenum* (96), and *Monoglobus pectinilyticus* (41).

### Degradation of type II arabinogalactan

Exo-β-1,3-galactanase (EC 3.2.1.145) cleaves the β-1,3-galactan backbone of type II arabinogalactan *via* bypassing the β-1,6-galactan side chains and releasing β-1,6-galactooligosaccharides and their derivatives (131). The β-1,6-galactan side chains are hydrolyzed to β-1,6-galactooligosaccharides of various degrees of polymerization (DP) by endo-β-1,6-galactanase (EC 3.2.1.164) (132, 133). On the other hand, exo-β-1,6-galactobiohydrolase releases β-1,6-galactobiose (β-1,6-Gal2) from the non-reducing terminal end of β-1,6-galactooligosaccharides, and α-L-arabinofuranosidase (EC 3.2.1.55) releases arabinofuranose (Araf) from α-1,3-Araf-substituted β-1,6-galactooligosaccharides (134). Exo-β-1,3-galactanase (Table 4) has been reported in *Monoglobus pectinilyticus* (41), *Bifidobacterium longum* subsp. *longum* (91), *Clostridium thermocellum* (97), *Phanerochaete chrysosporium* (98), *Sphingomonas sp*. (99), *Bacteroides thetaiotaomicron* (100), *Bacteroides ovatus* (100), *Bacteroides caccae* (101), and *Bacteroides cellulosilyticus* (101). Endo-β-1,6-galactanase (Table 4) had been reported in *Streptomyces avermitilis* NBRC14893 (102), *Bacteroides ovatus* (100), *Bacteroides caccae* (101), *Bacteroides cellulosilyticus* (101), and *Bifidobacterium longum* subsp. *longum* (91). Exo-β-1,6-galactobiohydrolase (Table 4) has been reported in *Monoglobus pectinilyticus* (41), *Bacteroides thetaiotaomicron* (101), *Streptomyces avermitilis* (102), *Bacteroides ovatus* (100), *Bacteroides caccae* (101), *Bacteroides cellulosilyticus* (101), and *Bifidobacterium longum* subsp. *longum* (91).

### Degradation of glucuronoxylan

Two enzymes, β-(1, 3–5)-D-xylan xylanohydrolase (endo-β-1,4 xylanase) (EC 3.2.1.8) and 1,4-β xylohydrolase (exo-β-1,4 xylanase) (EC 3.2.1.37), are involved in degrading the β-1,4 xylosyl linkages in unsubstituted domains along the xylan backbone of glucuronoxylan (GX) (135, 136). Glucuronoxylanase cleaves glucuronosyl moietes which are substituted as monomeric side chains on the xylan backbone (137). Endo-β-1,4-xylanase (Table 4) has been reported in *Pseudomonas boreopolis* G22 (103), *Bacteroides ovatus* (82), *Monoglobus pectinilyticus* (41), *Bacteroides thetaiotaomicron* (100), *Bacteroides caccae* (101), *Bacteroides cellulosilyticus* (101), *Clostridium thermocellum* (104), *Bacillus subtilis* (105), and *Streptomyces turgidiscabies* (106). Exo-β-1,4 xylanase (Table 4) has been reported in *Monoglobus pectinilyticus* (41), *Luteimicrobium* xylanilyticum (107), *Amycolatopsis mediterranei* (107), *Clostridium thermocellum* (104), *Bacillus subtilis* (105), and *Streptomyces turgidiscabies* (106).

### Degradation of xyloglucan

A set of glucanases and glycosidases are involved in cleaving xyloglucan (XG) into monosaccharides by two-step degradation (138, 139). Endo-β-1,4-glucanase (EC 3.2.1.4) hydrolyzes XG into large fragments, which are further degraded into monosaccharides by α-xylosidase (EC 3.2.1.177) and β-glucosidase (EC 3.2.1.21) (140). Endo-β-1,4-glucanase (Table 4) has been reported in *Monoglobus pectinilyticus* (41), *Caldicellulosiruptor kronotskyensis* (108), *Roseburia* sp. (42), *Eubacterium rectale* group (42), *Ruminococcus champanellensis* (109), *Ruminococcus bromii* (110), *Ruminiclostridium cellulolyticum* (111), and *Phaeoacremonium minimum* (112). Exo-β-1,4 xylanase (Table 4) has been reported in *Monoglobus pectinilyticus* (41), *Luteimicrobium xylanilyticum* (107), *Amycolatopsis mediterranei* (107), *Clostridium thermocellum* (104), *Bacillus subtilis* (105), *Streptomyces turgidiscabies* (106), *Ruminiclostridium cellulolyticum* (111), and *Phaeoacremonium minimum* (112). While α-D-xylosidase (Table 4) has been reported in *Sulfolobus solfataricus* P2 (39), *Talaromyces thermophilus* (113), *Cellvibrio japonicus* (114), *Bacteroides thetaiotaomicron* (82), *Bacteroides ovatus* (82), and *Monoglobus pectinilyticus* (41). Moreover, β-glucosidase (Table 4) has been reported in *Bifidobacterium adolescentis* (115), *Bacteroides ovatus* (116), *Listeria innocua* (117), *Streptomyces venezuelae* (118), *Pyrococcus furiosus* (119), *Cellvibrio japonicus* (114), *Caldicellulosiruptor saccharolyticus* (120), *Microbispora bispora* (121), *Thermoanaerobacter brockii* (122), *Thermobifida fusca* (103), *Pseudomonas* sp. (123), *Monoglobus pectinilyticus* (41), *Ruminococcus champanellensis* (109), and *Ruminococcus bromii* (110).

### Degradation of cellulose

The enzymes of the cellulase system consist of endo-β-1,4-glucanase (EC 3.2.1.4), exo-β-1,4-glucanase (EC 3.2.1.91), and β-glucosidase (EC 3.2.1.21). Endo-β-1,4-glucanase and exo-β-1,4-glucanase cleave cellulose to cellodextrins and cellobiose, which are then degraded to glucose by β-glucosidase (141, 142). Endo and exo-β-1,4-glucanase (Table 4) have been reported in *Monoglobus pectinilyticus* (41), *Caldicellulosiruptor kronotskyensis* (108), *Roseburia* sp. (42), *Eubacterium rectale* group (42), *Ruminococcus champanellensis* (109), *Ruminococcus* bromii (110), *Ruminiclostridium cellulolyticum* (111), *Paenibacillus* sp. (143), and *Phaeoacremonium minimum* (112). The enzyme β-glucosidase (Table 4) has been reported in *Bifidobacterium adolescentis* (115), *Bacteroides ovatus* (116), *Listeria innocua* (117), *Streptomyces venezuelae* (118), *Pyrococcus furiosus* (119), *Cellvibrio japonicus* (114), *Caldicellulosiruptor saccharolyticus* (120), *Microbispora bispora* (121), *Thermoanaerobacter brockii* (122), *Thermobifida fusca* (103), *Pseudomonas* sp. (123), *Monoglobus pectinilyticus* (41), *Ruminococcus champanellensis* (109), and *Ruminococcus bromii* (110).

## Processing to increase use of recalcitrant fibers

Our previous studies have already shown that physical processing technologies cannot significantly increase fiber degradability in monogastric animals (19, 144, 145). However, the utilization of enzymes added to feed is a promising method to improve fiber fermentability.

### Enzymatic and chemical modification

Supplementation of cell wall degrading enzymes to improve feed efficiency for pigs gets more and more attention from the feed industry nowadays. Supplementation of cell wall degrading enzymes can remove side-chains of polysaccharides in plant cell wall, which make them more accessible to bacterial enzymes (145). In addition, cell wall degrading enzymes are able to reduce the digesta viscosity, which might affect absorption of other nutrients, by cleaving the viscous polysaccharides (e.g., pectin). In the end, carbohydrases can depolymerize polysaccharides to oligosaccharides, which have potential prebiotic effects on the gut microbiota, leading to the health benefits for the host animal.

A previous study showed that pre-processing with sulfuric acid could increase utilization of carbohydrates from lignocellulosic biomass (146). Mild acid-treated rye (together with heat treatment) was reported to improve the release of arabinosyl residues in chickens (147). Alkaline pretreatment of rapeseed meal in the feed improved its fermentation in pigs (7). Therefore, chemical treatment in feed might be a promising method to improve fiber fermentation in pigs.

We have shown recently that RSM processed by enzymatic and chemical treatment led to modulation of the gut microbiota using a newly developed *in vitro* swine large intestinal model (SLIM) (148). In brief, our studies demonstrated that both enzymatic (cellulase or 2 different pectinases) and chemical (6 N sodium hydroxide) pretreatment on RSM shifted its cell wall polysaccharide structure, subsequently altering microbial community composition and functional profile compared to untreated RSM, and eventually increased fiber degradability as evaluated by SCFA production in SLIM. Moreover, it was validated in pigs, by the mobile nylon bag technique, that cellulase and alkaline treatment on RSM improved the overall degradation of RSM (149–151).

### Potential emerging technologies

High hydrostatic pressure, a non-thermal pasteurization technology, is applied to physical modification of chemical structure of compound (e.g., polysaccharides). High hydrostatic pressure could promote the water into interior of the matrix. As a result, high pressure (100–1,000 MPa) leads to the destruction of cell wall matrix (affecting non-covalent bonds, including hydrophobic interactions, hydrogen bonds, and van der Waals forces) and reduce substrate particle size. Research demonstrated that pectin (extracted from potato peel waste) could increase galacturonic acid content and decrease the esterification degree of pectin after high hydrostatic pressure at 200 MPa for 5 min (152). Another study reported that high hydrostatic pressure could significant reduce the molecular weight, degree of esterification and degree of acylation of sugar beet pectin at 250–500 MPa for 30 min (153). These reports have shown that high hydrostatic pressure could modify the polysaccharide structure, which might have a potential application value in improving fiber degradability of RSM in monogastric animals.

Cold plasma (CP) processing is a technology that causing the ionization of neutral gas (argon, helium, and nitrogen) and generating active species (free radicals, electrons, ions), which could induce oxidative damage in the cell wall compounds, such as polysaccharides. Therefore, CP could change structure and properties of polysaccharides. Study showed that CP could increase the viscosity and enhance the emulsion stabilization capacity of xanathan gum (154). Prasertsung et al. (155) reported that CP could degrade starches and cellulose into sugars and glucose (155). Therefore, CP might modify polysaccharides of RSM by inducing oxidative damage in its cell wall polysaccharides.

## Conclusions and perspectives

Rapeseed meal is not only a promising protein ingredient but also a potentially energy source for non-ruminant animal diets. RSM contains a high amount of cell wall polysaccharides, even higher when compared to soybean meal commonly used in the feed industry. However, information on degradation and utilization of RSM carbohydrates upon feeding these diets is insufficient, and for this it is essential to understand the characterization and quantification of RSM carbohydrates. For the sake of improving the degradation of RSM carbohydrates, it is necessary to understand the mode of actions of RSM polysaccharide degradation by microbes, discover factors that limit their utilization and absorption in the GIT, and develop strategies to improve efficiency and productivity. The use of exogenous enzymes, carbohydrases, becomes a good solution to improve RSM carbohydrate degradation, and eventually to improve animal digestibility, feed efficiency, and animal performance.

The current review summarized the polysaccharide types of RSM, gut microbiota which could degrade the polysaccharides, and glycoside hydrolases which used by the microorganism. The information offered above could be used to develop novel engineering microbes and enzymic preparations to improve RSM utilization in the future studies. One possible way is to deep mine the genomes of theses microbes for encoded the carbohydrases by bioinformatic tools. What is more, these enzymes can be overexpressed in an expression host and subsequently used to process RSM to investigate its degradability in livestock.

## Author contributions

CL prepared the original draft and edited the manuscript. X-LQ and KV critically reviewed the manuscript. All authors listed have approved the final manuscript for publication.

## Funding

This research has been partly made possible with the support of the Dutch Province of Limburg, with a grant to the Centre for Healthy Eating and Food Innovation (HEFI) of Maastricht University—Campus Venlo to KV. This study was also supported by the Young Teachers' Research and Innovation Ability Improvement Program of Beijing University of Agriculture (QJKC2022026), Technology Innovation spark action support program of Beijing University of Agriculture, and 2022 Beijing Municipal Education Commission Classification Development Project.

## Conflict of interest

The authors declare that the research was conducted in the absence of any commercial or financial relationships that could be construed as a potential conflict of interest.

## Publisher's note

All claims expressed in this article are solely those of the authors and do not necessarily represent those of their affiliated organizations, or those of the publisher, the editors and the reviewers. Any product that may be evaluated in this article, or claim that may be made by its manufacturer, is not guaranteed or endorsed by the publisher.
